# Nationwide Outcomes of Octogenarians Following Open or Endovascular Management After Ruptured Abdominal Aortic Aneurysms

**DOI:** 10.1177/15266028221083460

**Published:** 2022-03-21

**Authors:** Anna J. Alberga, Jorg L. de Bruin, Frederico Bastos Gonçalves, Eleonora G. Karthaus, Janneke A. Wilschut, Joost A. van Herwaarden, Jan J. Wever, Hence J. M. Verhagen

**Affiliations:** 1 Department of Vascular Surgery, Erasmus University Medical Center, Rotterdam, The Netherlands; 2 Scientific Bureau, Dutch Institute for Clinical Auditing, Leiden, The Netherlands; 3 Serviço de Angiologia e Cirurgia Vascular, Centro Hospitalar Universitário de Lisboa Central, NOVA Medical School, Lisboa, Portugal; 4 Department of Surgery, Amsterdam University Medical Center, Amsterdam, The Netherlands; 5 Department of Vascular Surgery, University Medical Centre Utrecht, Utrecht, The Netherlands; 6 Department of Vascular Surgery, Haga Teaching Hospital, The Hague, The Netherlands

**Keywords:** abdominal aortic aneurysm, rupture, endovascular aneurysm repair, open surgical repair, registry

## Abstract

**Purpose:**

Octogenarians are known to have less-favorable outcomes following ruptured abdominal aortic aneurysm (rAAA) repair compared with their younger counterparts. Accurate information regarding perioperative outcomes following rAAA-repair is important to evaluate current treatment practice. The aim of this study was to evaluate perioperative outcomes of octogenarians and to identify factors associated with mortality and major complications after open surgical repair (OSR) or endovascular aneurysm repair (EVAR) of a rAAA using nationwide, real-world, contemporary data.

**Methods:**

All patients that underwent EVAR or OSR of an infrarenal or juxtarenal rAAA between January 1, 2013, and December 31, 2018, were prospectively registered in the Dutch Surgical Aneurysm Audit (DSAA) and included in this study. The primary outcome was the comparison of perioperative outcomes of octogenarians versus non-octogenarians, including adjustment for confounders. Secondary outcomes were the identification of factors associated with mortality and major complications in octogenarians.

**Results:**

The study included 2879 patients, of which 1146 were treated by EVAR (382 octogenarians, 33%) and 1733 were treated by OSR (410 octogenarians, 24%). Perioperative mortality of octogenarians following EVAR was 37.2% versus 14.8% in non-octogenarians (adjusted OR=2.9, 95% CI=2.8–3.0) and 50.0% versus 29.4% following OSR (adjusted OR=2.2, 95% CI=2.2–2.3). Major complication rates of octogenarians were 55.4% versus 31.8% in non-octogenarians following EVAR (OR=2.7, 95% CI=2.1–3.4), and 68% versus 49% following OSR (OR=2.2, 95% CI=1.8–2.8). Following EVAR, 30.6% of the octogenarians had an uncomplicated perioperative course (UPC) versus 49.5% in non-octogenarians (OR=0.5, 95% CI=0.4–0.6), while following OSR, UPC rates were 20.7% in octogenarians versus 32.6% in non-octogenarians (OR=0.5, 95% CI=0.4–0.7). Cardiac or pulmonary comorbidity and loss of consciousness were associated with mortality and major complications in octogenarians. Interestingly, female octogenarians had lower mortality rates following EVAR than male octogenarians (adjusted OR=0.7, 95% CI=0.6–0.8).

**Conclusion:**

Based on this nationwide study with real-world registry data, mortality rates of octogenarians following ruptured AAA-repair were high, especially after OSR. However, a substantial proportion of these octogenarians following OSR and EVAR had an uneventful recovery. Known preoperative factors do influence perioperative outcomes and reflect current treatment practice.

## Introduction

A ruptured abdominal aortic aneurysm (rAAA) represents a highly lethal condition, especially in older patients. In patients undergoing surgical treatment for a rAAA, 30-day mortality rates of 24.5% (95% CI=23.4–25.7) following endovascular aneurysm repair (EVAR) and 37.8% (95% CI=36.4–39.2) following open surgical repair (OSR) are described.^1^ Advanced age is associated with increased in-hospital mortality.^2,3^ A meta-analysis which included studies up to 2010 reported perioperative mortality rates of 59.2% for octogenarians treated with OSR after rAAA^4^ and a more recent meta-analysis which included studies from centers of excellence, (nationwide) vascular registries, an administrative database, and an insurance database published 30-day mortality rates of 27% (95% CI=18–38) for octogenarians after EVAR and 52% (95% CI=44–60) after OSR, respectively.^5^ Although these perioperative mortality rates are high, AAA repair is the only option for these patients to survive a rAAA. Long-term outcomes of octogenarians who were successfully treated appeared to be reasonable as in the Swedish Vascular Registry, the survival of octogenarians treated for rAAAs and who survived the first 90 days after surgery was similar to non-octogenarians.^6^ Furthermore, in the recent meta-analysis, 1-year mortality rates in octogenarians of 35% (95% CI=18–56) following EVAR and 54% (95% CI=47–60) following OSR were reported.^5^

A swift decision regarding treatment is vital in patients with rAAAs. Current predictive models were developed using data up to 2012 and have limited value in predicting mortality or major complications following rAAA repair.^7-9^ However, this information is essential for decision-making regarding treatment or palliation, especially in elderly patients. The identification of patient factors associated with perioperative mortality in a nationwide cohort of octogenarians could evaluate the current selection of octogenarians to be treated for a rAAA. However, specific contemporary perioperative outcomes in octogenarians following rAAA repair based on a nationwide data reflecting real-world practice are scarce.

This study aimed to evaluate perioperative outcomes of octogenarians compared with non-octogenarians after OSR or EVAR of a ruptured infrarenal or juxtarenal AAA using nationwide, real-world, contemporary data. Furthermore, we identified factors associated with mortality and major complications in octogenarians and assessed time-trends of applied surgical techniques. Finally, we investigated the impact of complications on mortality and length of hospital stay based on a validated nationwide and mandatory clinical registry.

## Materials and Methods

### Data sources and Study Design

Data were collected from the Dutch Surgical Aneurysm Audit (DSAA), a mandatory nationwide clinical registry. All Dutch vascular surgeons performing aortic aneurysm interventions register their aortic aneurysm interventions in the DSAA. Since the establishment of the DSAA in 2013, the DSAA includes all patients that underwent repair of an infrarenal or juxtarenal aneurysm without previous aortic surgery and, thus, all rAAA repairs performed in the Netherlands were included in the DSAA. Data verification took place through a random sample of hospitals, concluding that the data had a high degree of reliability, with very few discrepancies detected and showing a case ascertainment of 98.4%.^10,11^ The data derived from this registry were anonymized and were retrospectively analyzed. The study followed the STROBE statement.^12^

### Participants

All consecutive patients that were registered in the DSAA undergoing primary repair (EVAR or OSR) of a ruptured infrarenal or juxtarenal AAA between January 1, 2013 and December 31, 2018 were included for analysis. Patients with missing date of birth, sex, or survival status at the time of discharge or 30-days postoperatively were excluded. No ethical approval or informed consent was required for this study according to Dutch law.

### Definitions

Age was calculated as year of surgery minus year of birth. Patients were considered octogenarians when their age was 80 years or older at the time of surgery. EVAR procedures followed by immediate conversion were categorized by intention-to-treat.

### Outcomes

The primary outcome was the comparison of perioperative outcomes (perioperative mortality, major complications, and the desirable composite outcome “uncomplicated perioperative course” [UPC]) of octogenarians with non-octogenarians. Secondary outcomes were the identification of factors associated with perioperative mortality and major complications in octogenarians and the influence of complications on perioperative mortality and length of hospital stay of living patients. Finally, time-trends per year regarding applied surgical techniques were evaluated.

Perioperative mortality was defined as death within 30-days or in-hospital. Major complications were defined as either intraoperative or postoperative complications that resulted in a prolonged length of stay, needed a reintervention, or caused mortality^13^ and described the perioperative period (first 30 days) following rAAA-repair. Prolonged length of stay was defined as the length of stay exceeding the 75th percentile of the length of stay of all living patients. The UPC was achieved when no perioperative mortality, no intraoperative complications, no postoperative surgical complications (for details, see Supplementary Table 1), no reinterventions, no readmission, and no prolonged length of stay occurred, and was based on the composite outcome Textbook Outcome, which was previously described for elective AAA repairs.^14^

### Statistical Methods

Baseline characteristics were stratified by EVAR and OSR and were compared between octogenarians and non-octogenarians. Categorical variables were compared between groups using Chi-square tests and Fisher exact tests, when appropriate. Continuous variables were compared using t-tests were used for normally distributed variables and Mann–Whitney U tests otherwise.

Differences in perioperative outcomes were examined with univariable logistic regression analyses with odds ratios including 95% confidence intervals. The associations between age ≥80 and mortality and major complications were examined for EVAR and OSR patients with multivariable logistic regression analyses using covariates and the factor “age ≥ 80.” In these analyses, patient characteristics based on both the V(p)-possum score^15^ and the Hardman index^16^ were included as covariates to adjust for confounding. Covariates used for analysis were gender, pulmonary comorbidity, cardiac comorbidity, abnormalities on preoperative electrocardiogram (ECG), preoperative renal dysfunction (creatine≥190μmol/L), systolic blood pressure (per 10mmHg), loss of consciousness (Glasgow Coma Scale<12), anemia (hemoglobin<5.6mmol/L), aneurysm diameter (per 10mm), and location of the aneurysm (abdominal aortic or aortoiliac). Factors with a p<0.10 in univariable analysis were selected for multivariable logistic regression analysis.

Factors associated with mortality and major complications were examined for octogenarians who underwent EVAR and OSR using logistic regression analyses. For this analysis, covariates mentioned earlier and age (as a continuous variable) were included. Factors with a p<0.10 in univariable analysis and factors considered clinically relevant (age and sex) were selected for multivariable logistic regression analysis.

Furthermore, we examined whether the proportion of applied surgical techniques and the proportion of octogenarians versus non-octogenarians decreased or increased linearly, using univariable logistic regression analyses. The impact of subgroups of complications, as registered in the DSAA, on mortality and median hospital-stay length of living patients was examined using descriptive statistics. For all analyses, statistical significance was defined as a p<0.05.

### Missing Data

The data of this subset from the DSAA contained variables with missing values (Supplementary Table 2). If patients with any missing data had been excluded from the multivariable analyses, information of 237 octogenarians (62%) that received EVAR and 290 octogenarians (71%) that received OSR would have been lost. In all variables with missing data, 7.8% and 10.1% of the information was missing for EVAR and OSR, respectively. No patterns of missing data were found. Therefore, missing data were assumed to be missing at random for all covariates allowing to impute missing data.^17^

In order to deal with the missing data, the method of multiple imputation using chained equations (MICE) was performed for both EVAR and OSR patients.^18^ Outcomes, as perioperative mortality, were not imputed. To account for the variation in completing the data set with multiple imputation, 60 data sets were used for EVAR patients and 70 data sets were used for OSR patients (each with 20 iterations).^18^ Further details are shown in Supplementary Table 2. After imputation, values that were imputed were compared with values that were observed using scatter plots and plots of the densities.

For the multivariable logistic regression models, the results of the imputed data sets were combined to produce a final result using the Rubin’s rules.^19^ For comparison, multivariable logistic regression models using the subsets of complete cases were performed.

All analyses were performed using R version 3.6.1.

## Results

**Figure 1.**
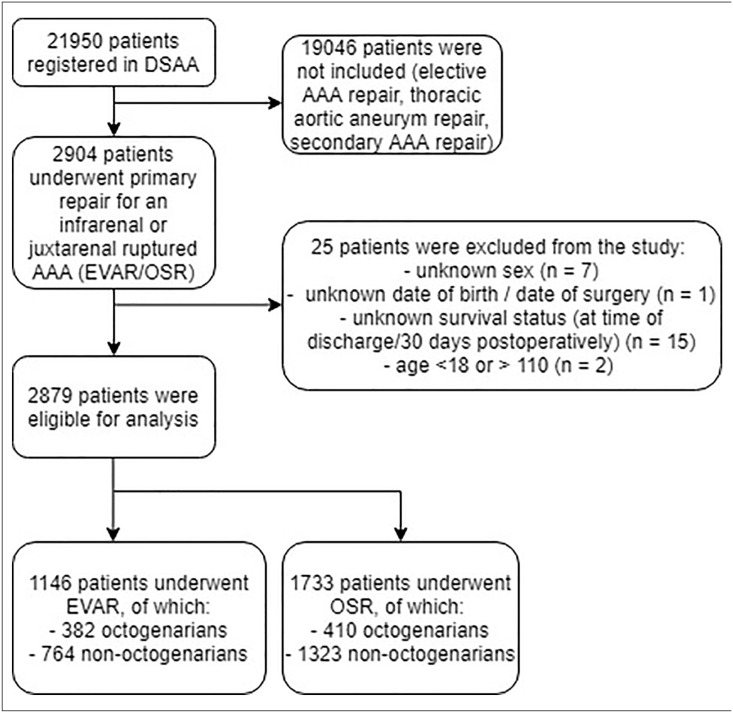
Flow diagram of included octogenarians and non-octogenarians following rAAA-repair. DSAA, Dutch Surgical Aneurysm Audit; EVAR, endovascular aneurysm repair; OSR, open surgical repair; rAAA, ruptured abdominal aortic aneurysm.

Between January 2013 and December 2018, 2904 patients from 61 hospitals who underwent primary repair for an infrarenal or juxtarenal rAAA were registered in the DSAA database. Of these patients, 2879 (99.1%) were eligible for analysis (Figure 1). In total, 792 octogenarians and 2087 non-octogenarians were included. Of all included patients, 1146 patients were treated with EVAR (382 octogenarians [33.3%] and 764 non-octogenarians (66.7%)), while 1733 patients were treated with OSR (410 octogenarians [23.7%] and 1323 non-octogenarians (76.3%)). In the EVAR group, 34 EVAR procedures (3.0%) were followed by immediate conversion. Of all 792 octogenarians in this study, 382 (48.2%) received EVAR, while of all 2087 non-octogenarians, 764 (36.6%) received EVAR (p<0.001).

### Patient Characteristics, Aneurysm Morphology, and Operative Data

Original data and percentages of imputed data of patient characteristics, aneurysm morphology, and operative data, are shown in Table 1. Octogenarians were more often female compared with non-octogenarians, especially in the OSR group. Furthermore, octogenarians had more cardiac comorbidities, pulmonary comorbidities, or abnormalities on ECG, and presented with lower baseline hemoglobin and higher baseline creatinine levels. Octogenarians undergoing EVAR and OSR more often had a loss of consciousness (GCS<12) compared with non-octogenarians. From 2013 up to 2015, the exact location of the aneurysm relative to the renal arteries and information of referral patterns were not registered in the DSAA. From 2016 to 2018, 210/750 (28.0%) AAAs registered following OSR were juxtarenal AAAs (46 octogenarians, 164 non-octogenarians) and 7/638 (1.1%) AAAs registered following EVAR had a juxtarenal location. Most patients were presented at the emergency department of the hospital in which they received treatment, 465/638 (72.9%) following EVAR, 586/791 (74.1%) following OSR, while some patients were first presented in another hospital, 60/638 (9.4%) following EVAR, 67/791 (8.5%) following OSR. It was not registered in the DSAA whether patients were suitable for EVAR. More procedural data (intraoperative blood loss, use of cell saver, intraoperative complications, admission to ICU, and length of ICU-stay) can be found in Supplementary Table 3.

**Table 1.** Patient Characteristics, Aneurysm Morphology and Operative Data for Octogenarians Versus Non-Octogenarians Undergoing EVAR and OSR.

	EVAR			OSR		
	<80	≥80	p	<80	≥80	p
Number of patients	764	382		1323	410	
Sex: female	87 (11.4) [11.4]	60 (15.7) [15.7]	<0.001 [0.049]	175 (13.2) [13.2]	106 (25.9) [25.9]	<0.001 [<0.001]
Age, years	70.33±6.69	84.52±3.40	N.A. [N.A.]	70.46±6.22	83.35±2.93	N.A. [N.A.]
Preoperative cardiac comorbidity			<0.001 [<0.001]			<0.001 [0.053]
None	337 (50.3) [44.1]	119 (36.3) [31.2]		581 (52.7) [43.9]	151 (47.1) [36.8]	
Medication for hypertension, angina pectoris, diuretics, or digoxin	276 (40.9) [36.1]	166 (49.9) [43.5]		431 (39.1) [32.6]	137 (43.0) [33.4]	
Peripheral edema, coumarins, borderline cardiomyopathy	44 (6.8) [5.8]	39 (12.0) [10.2]		74 (6.7) [5.6]	26 (8.2) [6.3]	
Elevated central venous pressure, cardiomegaly	12 (2.0) [1.6]	6 (1.9) [1.6]		15 (1.5) [1.1]	5 (1.7) [1.2]	
Unknown/missing	95 (–)[12.4]	52 (–) [13.6]		222 (–) [16.8]	91 (–) [22.2]	
Preoperative pulmonary comorbidity			<0.001 [0.002]			<0.001 [0.006]
No dyspnea	504 (77.5) [66.0]	205 (67.7) [53.7]		810 (79.4) [61.2]	219 (72.5) [53.4]	
Dyspnea during exercise	105 (16.8) [13.7]	71 (24.3) [18.6]		159 (16.0) [12.0]	70 (23.4) [17.1]	
Invalidating dyspnea	19 (3.1) [2.5]	13 (4.6) [3.4]		25 (2.9) [1.9]	9 (3.1) [2.2]	
Dyspnea at rest, consolidation, fibrosis	16 (2.6) [2.1]	8 (3.3) [2.1]		17 (1.7) [1.3]	1 (1.0) [0.2]	
Unknown/missing	120 (–) [15.7]	85 (–) [22.3]		312 (–) [23.6]	111 (–) [27.1]	
Preoperative ECG			<0.001 [<0.001]			<0.001 [0.005]
No abnormalities	259 (52.2) [33.9]	91 (36.2) [23.8]		403 (51.3) [30.5]	104 (46.0) [25.4]	
Atrial fibrillation	29 (6.0) [3.8]	40 (17.4) [10.5]		47 (6.6) [3.6]	31 (13.7) [7.6]	
Ischemia	26 (5.3) [3.4]	12 (5.2) [3.1]		45 (6.8) [3.4]	11 (6.1) [2.7]	
Other deviating results	186 (36.5) [24.3]	104 (41.1) [27.2]		271 (35.2) [20.5]	83 (34.2) [20.2]	
Not performed/missing	264 (–) [34.6]	135 (–) [35.3]		557 (–) [42.1]	181 (–) [44.1]	
Preoperative hemoglobin, mmol/L	7.40±1.40	6.90±1.30	<0.001 [<0.001]	7.40±1.39	6.85±1.34	<0.001 [<0.001]
Preoperative hemoglobin, mmol/L			<0.001 [0.172]			<0.001 [0.017]
≥ 5.6	657 (89.1) [86.0]	315 (85.0) [82.5]		1136 (89.9) [85.9]	334 (84.8) [81.5]	
< 5.6	81 (10.9) [10.6]	55 (15.0) [14.4]		125 (10.1) [9.4]	59 (15.2) [14.4]	
Unknown/missing	26 (–) [3.4]	12 (–) [3.1]		62 (–) [4.7]	17 (–) [4.1]	
Preoperative creatinine, μmol/L	106 [85.75–129.25]	116.00 [92.00–146.00]	<0.001 [<0.001]	106.00 [86.00–131.00]	113.00 [90.00–138.00]	0.001 [<0.001]
Preoperative creatinine, μmol/L			<0.001 [0.459]			0.240 [0.629]
up to 189	655 (90.9) [85.7]	317 (88.5) [83.0]		1132 (93.3) [85.6]	358 (93.5) [87.3]	
≥190	65 (9.1) [8.5]	40 (11.5) [10.5]		79 (6.7) [6.0]	23 (6.5) [5.6]	
Unknown/missing	44 (–) [5.8]	25 (–) [6.5]		112 (–) [8.5]	29 (–) [7.1]	
Preoperative systolic blood pressure, mmHg	110 [89.00 –135.0]	109 [85.50–130.00]	<0.001 [0.201]	106.00 [82.00–135.00]	100.00 [80.00–124.00]	<0.001 [0.007]
Unknown/missing	55 (–) [7.2]	31 (–) [8.1]		134 (–) [10.1]	44 [-] (10.7)	
Preoperative GCS			<0.001 [0.007]			0.001 [0.899]
Normal GCS (GCS≥12)	671 (95.9) [87.8]	318 (91.3) [83.2]		1021 (87.3) [77.2]	314 (86.6) [76.6]	
Loss of consciousness (GCS<12)	26 (4.1) [3.4]	29 (8.7) [7.6]		141 (12.7) [10.7]	47 (13.4) [11.5]	
Unknown/missing	67 (–) [8.8]	35 (–) [9.2]		161 (–) [12.2]	49 (–) [12.0]	
Location			<0.001 [0.578]			<0.001 [0.008]
Abdominal	718 (94.0) [94.0]	355 (91.3) [91.3]		1269 (95.9) [95.9]	404 (98.5) [98.5]	
Aortoiliac	46 (6.0) [6.0]	27 (7.1) [7.1]		54 (4.1) [4.1]	6 (1.5) [1.5]	
Diameter	76.37±17.11	75.35±17.40	<0.001 [0.362]	79.25±17.25	79.03±18.73	0.222 [0.833]
Unknown/missing	41 (–) [5.4]	33 (–) [8.6]		109 (–) [8.2]	35 (–) [8.5]	

Abbreviations: EVAR, endovascular aneurysm repair; GCS, Glasgow Coma Scale; OSR, open surgical repair.

Data are presented as n (%) and for continuous variables as mean±standard deviation (SD) or median with IQR Values in parentheses “(%)” are percentages after multiple imputation (60 data sets for EVAR patients, 70 data sets for OSR patients). Values in square brackets “[%]” are percentages including missing data. p-values without parentheses are calculated from data after multiple imputation and p-values in square brackets “[%]” are calculated from data including missing data.

### Perioperative Mortality

The perioperative mortality rate of all octogenarians was 43.8% compared with 24.1% in all non-octogenarians (p<0.001). Table 2 shows that octogenarians following EVAR had a mortality rate of 37.2% compared with 14.8% in non-octogenarians (OR=3.41, 95% CI=2.56–4.55). The mortality rate of octogenarians following OSR was 50.0% compared with 29.4% in non-octogenarians (OR=2.40, 95% CI=1.91–3.01).

**Table 2.** Perioperative Outcomes of Octogenarians Versus Non-Octogenarians After Both EVAR and OSR.

	EVAR					OSR				
	<80 (ref.)	≥80	OR (95% CI)^a^	OR (95% CI)^b^	aOR (95% CI)^c^	<80 (ref.)	≥80	OR (95% CI)^a^	OR (95% CI)^b^	aOR (95% CI)^c^
Number of patients	764	382				1323	410			
Perioperative mortality (intraoperative, 30-day and in-hospital)	113 (14.8)	142 (37.2)	3.41 (2.56–4.55)	3.41 (3.28–3.54)	2.87 (2.76–2.99)	389 (29.4)	205 (50.0)	2.40 (1.91–3.01)	2.40 (2.34–2.47)	2.21 (2.15–2.28)
Major complications^d^	242 (31.8)	211 (55.4)	2.66 (2.07–3.43)			652 (49.3)	279 (68.2)	2.21 (1.75–2.80)		
Uncomplicated perioperative course	378 (49.5)	117 (30.6)	0.45 (0.35–0.58)			431 (32.6)	85 (20.7)	0.54 (0.41–0.70)		
- No perioperative mortality	651 (85.2)	240 (62.8)				934 (70.6)	205 (50.0)			
- No complications during surgery	679 (88.9)	310 (81.2)				1078 (81.5)	308 (75.1)			
- No surgical complications	591 (77.4)	262 (68.6)				782 (59.1)	239 (58.3)			
- No reintervention	646 (84.6)	327 (85.6)				1024 (77.4)	322 (78.5)			
- No readmission	650 (85.1)	334 (87.4)				1152 (87.1)	354 (86.3)			
- No prolonged stay	585 (76.6)	289 (75.7)				1052 (79.5)	323 (78.8)			

Data are presented as n (%).

Abbreviations: CI, confidence interval; EVAR, endovascular aneurysm repair; OR, odds ratio; OSR, open surgical repair.

a OR using original data.

b OR using original data completed by multiple imputation.

c aOR using original data completed by multiple imputation, adjusted for gender, age≥80, cardiac comorbidity, pulmonary comorbidity, abnormalities on ECG, creatinine≥190, systolic blood pressure (per 10mmHg), hemoglobin<5.6, aortoiliac location, diameter (per 10mm).

d Major complication: post-operative death or a peri- or postoperative complication leading to a re-intervention or prolonged hospital stay (EVAR > 13 days, OSR > 24 days).

Moreover, after adjustment for confounders, octogenarians had a significantly higher mortality rate compared with non-octogenarians following both EVAR and OSR (EVAR: aOR 2.87, 95%-CI 2.76-2.99, OSR: aOR 2.21, 95%-CI 2.15–2.28). We found similar results using the subsets with complete cases following both EVAR (aOR 2.93, 95%-CI 1.74–4.98, p<0.001) and OSR (aOR 2.20, 95%-CI 1.39–3.48).

### Major Complications and Uncomplicated Perioperative Course

Table 2 shows that octogenarians develop major complications more often, compared with non-octogenarians, following both EVAR (55.4% vs 31.8%, OR=2.66, 95% CI=2.07–3.43) and OSR (68.2% vs 49.3%, OR=2.21, 95% CI=1.75–2.80). In octogenarians who underwent EVAR and developed major complications, cardiac complications were most common (28.9%), while abdominal complications were most common in octogenarians following OSR (28.7%). More details of cardiac and abdominal complications are provided in Supplementary Table 4. Octogenarians with major complications that survived perioperatively had a median length of hospital stay of 18 days (IQR=15–31) following EVAR and 31 days (IQR=26–41) following OSR (Table 3). Furthermore, octogenarians had less often an UPC than non-octogenarians following both EVAR (30.6% vs 49.5%, OR=0.45, 95% CI=0.35–0.58) and OSR (20.7% vs 32.6% (OR=0.54, 95% CI=0.41–0.70).

**Table 3.** Perioperative Outcomes (Perioperative Mortality and Length of Hospital-Stay of Living Patients) of All Octogenarians, Octogenarians With No Postoperative Complications, Octogenarians With Specific Postoperative Complications, Octogenarians With Major Complications, Stratified for EVAR and OSR.

	No. of octogenariansn (%)	Perioperative mortality n (%)	Length of hospital-stay of living patients median (IQR)
EVAR			
All octogenarians	382 (100)	142 (37.2)	9 [6–15]
Octogenarians with no postoperative complications	132 (34.6)	8 (6.1)	7 [5–10]
Octogenarians with 1 or more postoperative complications	249 (65.2)	133 (53.4)	13 [8–21]
Octogenarians with specific postoperative complications			
Abdominal	53 (13.9)	43 (81.1)	26.5 [18.75–33.50]
Cardiac	68 (17.8)	47 (69.1)	15 [6–18.5]
Pulmonary	73 (19.1)	27 (37.0)	15 [11–27.5]
Arterial occlusion	22 (5.8)	13 (59.1)	25 [18–32]
Reconstruction	20 (5.2)	8 (40.0)	6.50 [4.75–10.50]
Re-bleeding	16 (4.2)	15 (93.8)	NA
Wound	8 (2.1)	1 (12.5)	8 [7–13.5]
Neurologic	28 (7.3)	12 (42.9)	16.5 [8.75–31.5]
Renal	42 (11.0)	28 (66.7)	17 [13–21]
Other	73 (19.1)	41 (56.2)	15 [9–21]
Octogenarians with no major complications	170 (44.5)	0 (0.0)	8 [5–10]
Octogenarians with major complications	211 (55.2)	142 (67.3)	18 [15–31]
OSR			
All octogenarians	410 (100)	205 (50.0)	19 [12–28]
Octogenarians with no postoperative complications	90 (22.0)	26 (28.9)	11.5 [7.25–16.75]
Octogenarians with 1 or more postoperative complications	319 (77.8)	178 (55.8)	21 [15.5–32]
Octogenarians with specific postoperative complications			
Abdominal	84 (20.5)	54 (64.3)	31.5 [19–39.75]
Cardiac	82 (20.0)	49 (59.8)	21 [17–30]
Pulmonary	85 (20.7)	28 (32.9)	22.5 [16.75–37.25]
Arterial occlusion	34 (8.3)	19 (55.9)	30 [22–40]
Reconstruction	3 (0.7)	3 (100.0)	NA
Re-bleeding	28 (6.8)	23 (82.1)	17 [16–19]
Wound	10 (2.4)	3 (30.0)	25 [20–30.75]
Neurologic	40 (9.8)	12 (30)	31 [20.25–37.5]
Renal	74 (18.0)	48 (64.9)	27 [17–38]
Other	106 (25.9)	57 (53.8)	21 [16–36]
Octogenarians with no major complications	130 (31.7)	0 (0.0)	14 [9–19]
Octogenarians with major complications	279 (68.0)	205 (73.5)	31 [26–41]

Abbreviations: EVAR, endovascular aneurysm repair; OSR, open surgical repair.

Data are presented as n (%) and for continuous variables as median with IQR. Patients can suffer from more than 1 postoperative complication simultaneously and thus can fall into more than 1 postoperative complication category. Thus, the perioperative outcomes can also be caused by another complication than by the complication of the reported group.

### Factors Associated With Perioperative Mortality and Major Complications in Octogenarians

**Figure 2.**
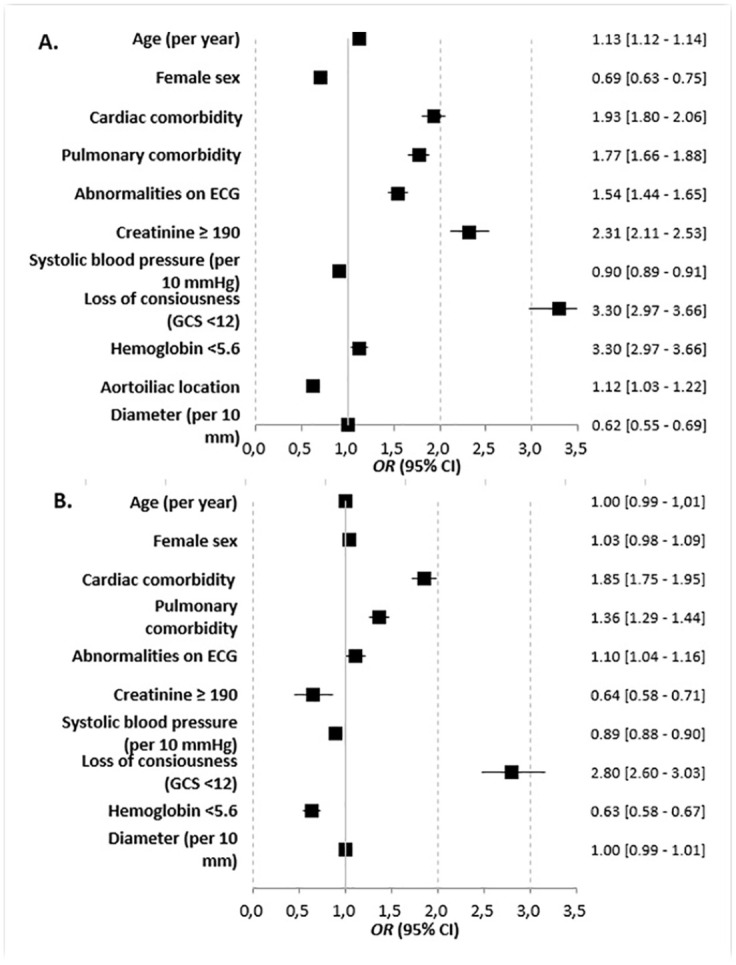
Forest plots showing the results of multivariable logistic regression analyses for EVAR (A) and OSR (B) to assess the association of patient-related risk factors with perioperative mortality in octogenarians (using original data completed by multiple imputation). OR>1 indicates higher mortality. EVAR, endovascular aneurysm repair; OSR, open surgical repair.

Figure 2 and Supplementary Table 5 show patient-related risk factors that are associated with mortality in octogenarians. Following EVAR, an increase in age was associated with mortality, while female sex was associated with less mortality. Following OSR, both an increase in age and gender were not associated with mortality. Moreover, creatinine ≥190 was associated with less mortality, as well as hemoglobin <5.6. Loss of consciousness, abnormalities on ECG, and pulmonary and cardiac comorbidity were associated with mortality in both groups. Analysis on a subgroup of patients to assess patient-related risk factors including aneurysm location (infrarenal or juxtarenal) in octogenarians that underwent OSR showed that a juxtarenal aneurysm location was associated with less mortality compared with an infrarenal location (aOR=0.93, 95% CI=0.89–0.98).

**Figure 3.**
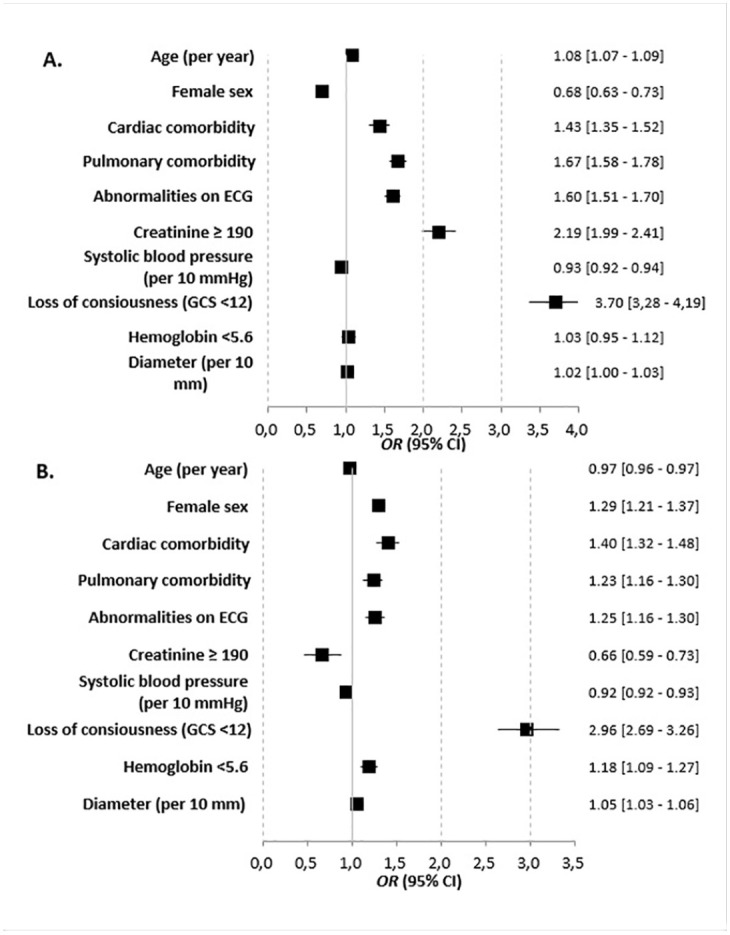
Forest plots showing the results of multivariable logistic regression analyses for EVAR (A) and OSR (B) to assess the association of patient-related risk factors with major complications in octogenarians (using original data completed by multiple imputation). OR>1 indicates more major complications. EVAR, endovascular aneurysm repair; OSR, open surgical repair.

Figure 3 and Supplementary Table 6 show that in octogenarians, an increase in age was associated with more major complications following EVAR, while female sex was associated with fewer major complications. Following OSR, an increase in age was associated with fewer complications, while female sex was associated with more major complications. Abnormalities on ECG, pulmonary, cardiac comorbidity, and loss of consciousness were associated with major complications in both groups.

### Time Trends of Applied Surgical Technique, Number of Hospitals, Perioperative Mortality and Proportion of Octogenarians

In all patients and in the subgroup of octogenarians, EVAR was increasingly applied compared with OSR (all patients: OR=1.15, 95% CI=1.10–1.21; octogenarians: OR=1.18, 95% CI=1.09–1.29). In all patients, the percentage of EVAR increased from 29% in 2013 to 47% in 2018, while in octogenarians, the percentage of EVAR increased from 39% in 2013 to 55% in 2018. In 2013, 36 hospitals performed EVAR in ruptured setting, while in 2018, 47 hospitals performed EVAR in ruptured setting. The number of hospitals that performed OSR in ruptured setting was 55 in 2013 and 52 in 2018. The perioperative mortality remained stable over the years in all patients, and in the subgroup of octogenarians (all patients: OR=0.98, 95% CI=0.94–1.03; octogenarians: OR=1.02, 95% CI=0.94–1.11), despite a higher proportion of patients treated with EVAR. During the study period, the proportion of octogenarians versus non-octogenarians was stable in all patients (OR=0.98, 95% CI=0.93–1.03), OSR patients (OR=0.95, 95% CI=0.89–1.02), and EVAR patients (OR=0.97, 95% CI=0.91–1.05).

### The Impact of Complications on Perioperative Outcomes in Octogenarians

Table 3 shows that some octogenarians with specific complications have high perioperative mortality rates (EVAR: abdominal complication 81.1%, re-bleeding 93.8%; OSR: re-bleeding 82.1%, and renal complication: 64.9%). Moreover, an abdominal complication or an arterial occlusion following EVAR, and an abdominal or a neurologic complication following OSR have the highest median length of hospital-stay of living patients.

## Discussion

This study showed that perioperative mortality rates of octogenarians following both EVAR and OSR were high (37.2% after EVAR; 50.0% after OSR) and significantly unfavorable compared with younger patients, similar to the existing literature.^2,4^ However, a substantial proportion of the octogenarians had an uneventful recovery after surgery (1/3 after EVAR and 1/5 after OSR). The preoperative risk factors pulmonary or cardiac comorbidity and loss of consciousness were associated with mortality and major complications in octogenarians following both EVAR and OSR for rAAAs, while an increased age was not associated with mortality following OSR, and female sex was associated with less mortality following EVAR.

The recently published ESVS guidelines state that acceptable results of treatment for rAAA can be achieved in patients aged >80 years.^20^ A recent meta-analysis regarding the outcome of rAAA-repair in octogenarians, which included besides a study from the nationwide Swedish Vascular Registry, studies from expert centers, (non-nationwide) vascular registries, an administrative database, and an insurance database, found pooled 30-day mortality rates of 27% (95% CI=18–38) following EVAR and 52% (95% CI=44–60)^5^ following OSR. Interestingly, our perioperative mortality rates following OSR were similar compared with the rates reported in the meta-analysis, while our perioperative mortality rates following EVAR were increased. In our study, “age ≥ 80” was associated with mortality following both EVAR (aOR=2.87) and OSR (aOR=2.21), while a recent nationwide study using administrative data from Japan reported that “age ≥ 80” was not associated with mortality in patients that underwent EVAR (aOR=1.13, 95% CI=0.77–1.66) and described mortality rates of 24.7% in octogenarians and 23.5% in younger patients following EVAR.^21^ Furthermore, 55% of the octogenarians did not undergo OSR or EVAR in this Japanese study. In contrast, a recent Dutch multicentre cohort study reported a turndown rate for rAAA treatment of only 29.9%,^22^ suggesting that the Japanese octogenarians were more strictly selected. Therefore, although we could not report the turndown rate of our nationwide cohort, we hypothesize that the increased perioperative mortality rates after EVAR in our study may suggest that relatively many high-risk octogenarians in the Netherlands underwent EVAR for rAAAs as a last resort.

Moreover, we described major complications and UPC rates in octogenarians compared with non-octogenarians. As expected, major complication rates were higher, and UPC rates were lower in octogenarians compared with non-octogenarians. The UPC was based on Textbook Outcome, which is usually used for reporting the Quality of Care in an elective setting of abdominal aneurysm treatment.^14^ We chose to describe UPC rates to clarify the proportion of octogenarians that achieves a desirable outcome following rAAA-repair, which was not described before. As is shown in Table 2, it is important to note that about 1/3 of octogenarians with rAAA undergoing EVAR and 1/5 undergoing ORS had a completely uneventful recovery, arguing that the current selection process for surgery is quite acceptable. Although we did not have information on turndown rates, better outcomes of octogenarians could probably be achieved with stricter selection for treatment. However, in our opinion, our UPC results suggest that turning down patients solely based on their age should be avoided since some octogenarians do have acceptable results. Besides, it is described that a substantial proportion of the octogenarians that survive rAAA repair (>80%) returned to their home after rehabiliaton.^22^ Moreover, it is hard to predict patients that definitely will perish after surgery, and it remains questionable whether current prediction models are sufficient to do this in this patient category reliably.^8^

Without an almost perfect prediction, patients will not be withheld from treatment using a scoring system.^7^ Although current prediction models, using a cohort with patients up to 2015, could not reliably predict mortality in preoperative setting with area under the receiver operating characteristic curves (AUCs) varying from 0.59 to 0.72,^7^ we did not develop a new predictive model since our database does not include patients turned down for surgery and lacks morphological or anatomical details. In 2016, the Dutch Aneurysm Score (DAS) was developed, which reported an externally validated AUC of 0.77.^9^ However, we could not externally validate the DAS using our nationwide cohort since the DSAA does not include information on cardiopulmonary resuscitation. Therefore, our study described preoperative patient risk factors associated with perioperative mortality and major complications in octogenarians, reflecting their current real-life treatment practice. Loss of consciousness was highly associated with mortality and major complications following both EVAR and OSR, suggesting that few octogenarians with loss of consciousness survive rAAA-repair. Interestingly, we found that females had lower mortality following EVAR, but not following OSR. Usually, higher 30-day mortality rates in women following rAAA-repair have been reported.^23^ Therefore, our results could suggest that female octogenarians potentially were strictly selected for EVAR. Moreover, we found that in both treatment groups, lower systolic blood pressure was associated with less mortality, which was not in line with another study that reported on predictors of mortality after repair of rAAAs.^24^ This somewhat contradictory result may be due to higher turndown rates for octogenarians with low systolic blood pressure. In octogenarians, increased age was associated with mortality following EVAR, but was not associated with mortality following OSR. Moreover, elevated preoperative creatinine and decreased preoperative hemoglobin, a predictor for mortality in the DAS,^9^ were associated with less mortality in octogenarians following OSR. In addition, a juxtarenal aneurysm location was associated with less mortality compared with an infrarenal location following OSR, which was in contrast with the results of the IMPROVE trial in which a short aneurysm neck of rAAAs was associated with mortality following OSR.^25^ We hypothesize that all these counterintuitive findings could be a reflection of selection bias and that octogenarians with elevated preoperative creatinine, decreased preoperative hemoglobin, or a juxtarenal aneurysm who received OSR were more strictly selected for surgery, resulting in a selection of relatively fit octogenarians with low general frailty. For Dutch vascular surgeons, this information reflects the current treatment practice of octogenarians. This information could serve as a first step to evaluate the selection of octogenarians for surgery. However, it will be essential to have additional information on octogenarians who were not selected for surgery to assess the entire selection process of octogenarians for surgery. Moreover, survival after aneurysm repair is not the sole parameter of clinical success and should be complemented by patient-centered outcomes such as health-related quality of life and postoperative living situation.^22^

This study shows that octogenarians underwent EVAR more frequently than non-octogenarians (48.2% vs 36.6%) and that EVAR was increasingly applied during the study period in both octogenarians and non-octogenarians. Our study showed that the endovascular treatment of rAAAs in all patients increased from 29% in 2013 to 47% in 2018. This percentage has increased over the years since physicians became more familiar with the EVAR technique and a stock of stent-grafts became more widely available.^26^ However, the actual clinical decision in octogenarians regarding the surgical procedure when both EVAR and OSR are suitable could be dependent on local or regional setting as well.^27^ Surprisingly, our study showed that despite the increased use of EVAR, the perioperative mortality rates of octogenarians and all patients were stable over the years, which was in contrast with previous studies.^1,28^ A possible explanation for these stable mortality rates could be that due to the increased application of EVAR, relatively more frail patients have received treatment for an rAAA during the study period, which could have influenced perioperative mortality rates.^29^ Although the proportion of octogenarians did not change over the years in our study, we could not objectify an increasing frailty rate with the data registered in the DSAA. The decreasing numbers of OSR in ruptured setting should have our attention in upcoming years, as a study that included data from 11 vascular registries reported lower in-hospital mortality rates in hospitals with high volumes of OSR in ruptured setting.^30^

For a correct interpretation of our findings, it is important to note that the results of OSR patients are not directly comparable with the results of EVAR patients since the characteristics of patients included in both groups were not similar. Octogenarians that underwent OSR had more often loss of consciousness and were more frequently female, compared with the octogenarians that underwent EVAR. It was described that in intact setting, only 34% of the female were morphologically suitable for EVAR within the instructions for use (IFU) due to short and angulated proximal aneurysm necks or unsuitable iliac arteries (access vessels) compared with 54% in male,^31^ which could clarify our high proportion of females undergoing OSR. Moreover, in our study, the OSR group included several juxtarenal AAAs, while only a few juxtarenal AAAs were treated with EVAR, which was probably due to a lack of suitable endovascular treatment options for juxtarenal AAAs. Future solutions with physician-modified grafts, chimney technique or off-the-shelf solutions might change these numbers.^32–34^ Besides the differences in measured characteristics, which could be a reflection of selection bias and could influence outcomes, unmeasured characteristics were probably also different between the two groups. Therefore, we refrained from a direct comparison between both groups (EVAR vs OSR) and propensity score matching.

A limitation of this study is that our data was retrieved from a nationwide quality registry that contains limited data. Consequently, we could not correct for all potential confounders in our multivariable analyses—for example, as mentioned before, we could not correct for frailty since frailty was not registered in our registry. Frailty is described as an independent predictor of in-hospital mortality in emergency general surgery^29^ and could influence perioperative mortality rates. Moreover, the variable aneurysm location (infrarenal or juxtarenal) was not available in the entire study period. Therefore, we could only perform an analyses on a subgroup of patients to assess the influence of aneurysm location on perioperative mortality. Finally, no information is available of patients not eligible for surgical repair since only patients who were stable enough to reach the hospital and underwent surgery were included. Therefore, our findings could be a reflection of selection bias and we could not report on all patients with rAAAs. An important strength of our study is the mandatory nationwide setup.

## Conclusion

This nationwide study provides us with valuable real-life contemporary data on outcomes after repair of rAAA in octogenarians that could serve as a first step to evaluate the selection of octogenarians for surgery. Although aneurysm repair is associated with high mortality in this patient category, especially after OSR, it is important to realize that a substantial proportion of these patients (1/3 after EVAR and 1/5 after OSR) had an uneventful recovery. Known preoperative risk factors do influence these outcomes and reflect current treatment practice.

## Supplemental Material

sj-docx-1-jet-10.1177_15266028221083460 – Supplemental material for Nationwide Outcomes of Octogenarians Following Open or Endovascular Management After Ruptured Abdominal Aortic Aneurysms Click here for additional data file. Supplemental material, sj-docx-1-jet-10.1177_15266028221083460 for Nationwide Outcomes of Octogenarians Following Open or Endovascular Management After Ruptured Abdominal Aortic Aneurysms by Anna J. Alberga, Jorg L. de Bruin, Frederico Bastos Gonçalves, Eleonora G. Karthaus, Janneke A. Wilschut, Joost A. van Herwaarden, Jan J. Wever and Hence J. M. Verhagen in Journal of Endovascular Therapy

sj-docx-2-jet-10.1177_15266028221083460 – Supplemental material for Nationwide Outcomes of Octogenarians Following Open or Endovascular Management After Ruptured Abdominal Aortic Aneurysms Click here for additional data file. Supplemental material, sj-docx-2-jet-10.1177_15266028221083460 for Nationwide Outcomes of Octogenarians Following Open or Endovascular Management After Ruptured Abdominal Aortic Aneurysms by Anna J. Alberga, Jorg L. de Bruin, Frederico Bastos Gonçalves, Eleonora G. Karthaus, Janneke A. Wilschut, Joost A. van Herwaarden, Jan J. Wever and Hence J. M. Verhagen in Journal of Endovascular Therapy

sj-docx-3-jet-10.1177_15266028221083460 – Supplemental material for Nationwide Outcomes of Octogenarians Following Open or Endovascular Management After Ruptured Abdominal Aortic Aneurysms Click here for additional data file. Supplemental material, sj-docx-3-jet-10.1177_15266028221083460 for Nationwide Outcomes of Octogenarians Following Open or Endovascular Management After Ruptured Abdominal Aortic Aneurysms by Anna J. Alberga, Jorg L. de Bruin, Frederico Bastos Gonçalves, Eleonora G. Karthaus, Janneke A. Wilschut, Joost A. van Herwaarden, Jan J. Wever and Hence J. M. Verhagen in Journal of Endovascular Therapy

sj-docx-4-jet-10.1177_15266028221083460 – Supplemental material for Nationwide Outcomes of Octogenarians Following Open or Endovascular Management After Ruptured Abdominal Aortic Aneurysms Click here for additional data file. Supplemental material, sj-docx-4-jet-10.1177_15266028221083460 for Nationwide Outcomes of Octogenarians Following Open or Endovascular Management After Ruptured Abdominal Aortic Aneurysms by Anna J. Alberga, Jorg L. de Bruin, Frederico Bastos Gonçalves, Eleonora G. Karthaus, Janneke A. Wilschut, Joost A. van Herwaarden, Jan J. Wever and Hence J. M. Verhagen in Journal of Endovascular Therapy

sj-docx-5-jet-10.1177_15266028221083460 – Supplemental material for Nationwide Outcomes of Octogenarians Following Open or Endovascular Management After Ruptured Abdominal Aortic Aneurysms Click here for additional data file. Supplemental material, sj-docx-5-jet-10.1177_15266028221083460 for Nationwide Outcomes of Octogenarians Following Open or Endovascular Management After Ruptured Abdominal Aortic Aneurysms by Anna J. Alberga, Jorg L. de Bruin, Frederico Bastos Gonçalves, Eleonora G. Karthaus, Janneke A. Wilschut, Joost A. van Herwaarden, Jan J. Wever and Hence J. M. Verhagen in Journal of Endovascular Therapy

sj-docx-6-jet-10.1177_15266028221083460 – Supplemental material for Nationwide Outcomes of Octogenarians Following Open or Endovascular Management After Ruptured Abdominal Aortic Aneurysms Click here for additional data file. Supplemental material, sj-docx-6-jet-10.1177_15266028221083460 for Nationwide Outcomes of Octogenarians Following Open or Endovascular Management After Ruptured Abdominal Aortic Aneurysms by Anna J. Alberga, Jorg L. de Bruin, Frederico Bastos Gonçalves, Eleonora G. Karthaus, Janneke A. Wilschut, Joost A. van Herwaarden, Jan J. Wever and Hence J. M. Verhagen in Journal of Endovascular Therapy
