# Supplementary material for: Nationwide Outcomes of Octogenarians Following Open or Endovascular Management After Ruptured Abdominal Aortic Aneurysms
Source: J Endovasc Ther. 2022 Mar 21;30(3):419–32. doi: 10.1177/15266028221083460 (PMC10209502; doi:10.1177/15266028221083460)
Supplement: sj-docx-1-jet-10.1177_15266028221083460 – Supplemental material for Nationwide Outcomes of Octogenarians Following Open or Endovascular Management After Ruptured Abdominal Aortic Aneurysms [file sj-docx-1-jet-10.1177_15266028221083460.docx]

**Supplementary Table 1**

The following complications were defined as a surgical complications:

- any abdominal complication (abdominal abscess, abdominal sepsis, ileus, spleen injury, bowel ischemia, bowel injury, stoma placement, other abdominal complications)

- any arterial occlusion ((major) amputation, renal artery arterial occlusion, other arterial occlusion (including trash foot))

- any prosthesis-/reconstruction related complication (prosthesis infection, prosthesis migration, other prosthesis-/reconstruction related complications)

- any wound complication (deep wound infection, fascia dehiscence, other wound complications)

- any renal complication (renal insufficiency (without hemodialysis or requiring hemodialysis))

- any rebleeding

- pulmonary complication: pneumothorax

- neurologic complication: paraplegia
