# Supplementary material for: Nationwide Outcomes of Octogenarians Following Open or Endovascular Management After Ruptured Abdominal Aortic Aneurysms
Source: J Endovasc Ther. 2022 Mar 21;30(3):419–32. doi: 10.1177/15266028221083460 (PMC10209502; doi:10.1177/15266028221083460)
Supplement: sj-docx-2-jet-10.1177_15266028221083460 – Supplemental material for Nationwide Outcomes of Octogenarians Following Open or Endovascular Management After Ruptured Abdominal Aortic Aneurysms [file sj-docx-2-jet-10.1177_15266028221083460.docx]

**Supplementary Table 2**

Variables used for multiple imputation, including the number of records for which each variable is missing and the imputation method that was used; for binary data logistic regresson was used and for unordered categorical data polytomous logistic regression (polyreg) was used. Bayesian linear regression (norm) was used for normal-distributed numeric data (norm), and data predictive mean meathing (pmm) was used for non-normal distributed numeric data^[[1]](#footnote-1)^.

|  | Missing - EVAR (n, (%) | Missing -  OSR (n, %) | Imputation method |
| --- | --- | --- | --- |
| Cardiac history | 147 (12.8) | 313 (18.1) | Polyreg* |
| Pulmonary history | 205 (17.9) | 423 (24.4) | Polyreg* |
| ECG abnormalities | 399 (34.8) | 738 (42.6) | Polyreg* |
| Hemoglobin | 38 (3.3) | 79 (4.6) | Norm** |
| Creatinine | 69 (6.0) | 141 (8.1) | Pmm*** |
| GCS | 102 (8.9) | 210 (12.1) | Polyreg* |
| Systolic blood pressure | 86 (7.5) | 178 (10.3) | Pmm*** |
| Diameter of the aneurysm | 74 (6.4) | 144 (8.3) | Norm** |
| Perioperative complications | 10 (0.9) | 7 (0.4) | Polyreg* |
| Length of stay at ICU | 12 (1.0) | 29 (1.7) | Pmm*** |
| Length of hospital stay | 27 (2.4) | 36 (2.1) | Pmm*** |
| Reinterventions | 3 (0.3) | 5 (0.3) | Logreg**** |
| Readmission | 77 (6.7) | 145 (8.4) | Logreg**** |
| Complications | 2 (0.2) | 3 (0.2) | Logreg**** |
| Also included as complete data: year of surgery, age, gender, location of the aneurysm, survival status and procedure |  |  |  |

* Polytomous logistic regression ** Bayesian linear regression *** Predictive mean matching **** Logistic regression

Datasets of EVAR and OSR patients were separately imputed. All variables were included in the multiple imputation as predictor, except the variables ‘Length of stay at ICU’ and ‘Length of hospital stay’ that were not included as predictor due to strong correlations.

In the complete dataset, 451 (39.4%) EVAR patients and 527 (30.4%) OSR patients had no missing data.

1. <https://www.rdocumentation.org/packages/mice/versions/3.10.0/topics/mice> [↑](#footnote-ref-1)
