# Supplementary material for: Nationwide Outcomes of Octogenarians Following Open or Endovascular Management After Ruptured Abdominal Aortic Aneurysms
Source: J Endovasc Ther. 2022 Mar 21;30(3):419–32. doi: 10.1177/15266028221083460 (PMC10209502; doi:10.1177/15266028221083460)
Supplement: sj-docx-3-jet-10.1177_15266028221083460 – Supplemental material for Nationwide Outcomes of Octogenarians Following Open or Endovascular Management After Ruptured Abdominal Aortic Aneurysms [file sj-docx-3-jet-10.1177_15266028221083460.docx]

**Supplementary Table 3**

|  | EVAR 80- (n = 764) | EVAR 80+ (n = 382) | P-value |
| --- | --- | --- | --- |
| Intraoperative blood loss |  |  | 0.206 |
| - 0-100 ml | 208 (27.2) | 82 (21.5) |  |
| - 101-500 ml | 271 (35.5) | 146 (38.2) |  |
| - 501-999 ml | 28 (3.7) | 18 (4.7) |  |
| - >1000 ml | 58 (7.6) | 37 (9.7) |  |
| - NA/missing | 199 (26.0) | 99 (25.9) |  |
| Use of cell saver* | 37/417 (8.9) | 24/221 (10.9) | 0.502 |
| Intraoperative complications |  |  | <0.001 |
| - No | 679 (88.9) | 310 (81.2) |  |
| - Cardiopulmonary resuscitation / myocardial infarction | 17 (2.2) | 28 (7.3) |  |
| - Other | 57 (7.5) | 40 (10.5) |  |
| - Death | 4 (0.5) | 1 (0.3) |  |
| - NA/missing | 7 (0.9) | 3 (0.8) |  |
| Admission to ICU |  |  | 0.932 |
| - No | 96 (12.6) | 51 (13.4) |  |
| - Yes | 660 (86.4) | 327 (85.6) |  |
| - NA/missing | 8 (1.0) | 4 (1.0) |  |
| Length of ICU-stay (median [IQR] | 1 [1, 3] | 1 [1, 3] | 0.664 |

** data from 2016 is shown*

|  | OSR 80- (n = 1323) | OSR 80+ (n = 410) | P-value |
| --- | --- | --- | --- |
| Intraoperative blood loss |  |  | **0.025** |
| - 0-100 ml | 5 (0.4) | 5 (1.2) |  |
| - 101-500 ml | 45 (3.4) | 18 (4.4) |  |
| - 501-999 ml | 92 (7.0) | 31 (7.6) |  |
| - >1000 ml | 899 (68.0) | 248 (60.5) |  |
| - NA/missing | 282 (21.3) | 108 (26.3) |  |
| Use of cell saver* |  |  | 0.062 |
| - No | 120/613 (19.6) | 48/178 (27.0) |  |
| - Yes | 492/613 (80.3) | 130/178 (73.0) |  |
| - NA/missing | 1/613 (0.2) | 0/613 (0.0) |  |
| Intraoperative complications |  |  | 0.005 |
| - No | 1078 (81.5) | 308 (75.1) |  |
| - Cardiopulmonary resuscitation / myocardial infarction | 82 (6.2) | 34 (8.3) |  |
| - Other | 154 (11.6) | 59 (14.4) |  |
| - Death | 4 (0.3) | 7 (1.7) |  |
| - NA/missing | 5 (0.4) | 2 (0.5) |  |
| Admission to ICU |  |  | 0.932 |
| - No | 68 (5.1) | 46 (11.2) |  |
| - Yes | 1231 (93.0) | 359 (87.6) |  |
| - NA/missing | 24 (1.8) | 5 (1.2) |  |
| Length of ICU-stay (median [IQR] | 3 [2, 8] | 3 [1, 8] | 0.664 |

** data from 2016 is shown*

|  | EVAR 80+, patients who survived perioperatively (n = 240) | EVAR 80+, patients who died perioperatively (n = 142) | P-value |
| --- | --- | --- | --- |
| Intraoperative blood loss |  |  | **0.001** |
| - 0-100 ml | 57 (23.8) | 25 (17.6) |  |
| - 101-500 ml | 102 (42.5) | 44 (31.0) |  |
| - 501-999 ml | 11 (4.6) | 7 (4.9) |  |
| - >1000 ml | 13 (5.4) | 24 (16.9) |  |
| - NA/missing | 57 (23.8) | 42 (29.6) |  |
| Use of cell saver* | 13/135 (9.6) | 11/86 (12.8) | 0.607 |
| Intraoperative complications |  |  | <0.001 |
| - No | 224 (93.3) | 86 (60.6) |  |
| - Cardiopulmonary resuscitation / myocardial infarction | 2 (0.8) | 26 (18.3) |  |
| - Other | 11 (4.6) | 29 (29.4) |  |
| - Death | 0 (0.0) | 1 (0.7) |  |
| - NA/missing | 3 (1.2) | 0 (0.0) |  |
| Admission to ICU |  |  |  |
| - No | 28 (11.7) | 23 (16.2) | 0.100 |
| - Yes | 221 (87.9) | 116 (81.7) |  |
| - NA/missing | 1 (0.4) | 3 (2.1) |  |
| Length of ICU-stay (median [IQR] | 1 [1, 3] | 1 [1, 3] | 0.939 |

** data from 2016 is shown*

|  | OSR 80+, patients who survived perioperatively (n = 205) | OSR 80+, patients who died perioperatively (n = 205) | P-value |
| --- | --- | --- | --- |
| Intraoperative blood loss |  |  | 0.054 |
| - 0-100 ml | 2 (1.0) | 3 (1.5) |  |
| - 101-500 ml | 10 (4.9) | 8 (3.9) |  |
| - 501-999 ml | 22 (10.7) | 9 (4.4) |  |
| - >1000 ml | 126 (61.5) | 122 (59.5) |  |
| - NA/missing | 45 (22.0) | 63 (30.7) |  |
| Use of cell saver* | 57/80 (71.2) | 73/98 (74.5) | 0.753 |
| Intraoperative complications |  |  | < 0.001 |
| - No | 178 (86.8) | 130 (63.4) |  |
| - Cardiopulmonary resuscitation / myocardial infarction | 1 (0.5) | 33 (16.1) |  |
| - Other | 25 (12.2) | 34 (16.6) |  |
| - Death | 0 (0.0) | 7 (3.4) |  |
| - NA/missing | 1 (0.5) | 1 (0.5) |  |
| Admission to ICU |  |  | < 0.001 |
| - No | 6 (2.9) | 40 (19.5)** |  |
| - Yes | 197 (96.1) | 162 (79.0) |  |
| - NA/missing | 2 (1.0) | 3 (1.5) |  |
| Length of ICU-stay (median [IQR] | 5 [3, 11] | 1 [1, 5] | <0.001 |

** data from 2016 is shown*

*** Of these patients: 21 patients received cardiopulmonary resuscitation or had a myocardial infarction during surgery, and 7 patients died during surgery.*
