# Supplementary material for: Nationwide Outcomes of Octogenarians Following Open or Endovascular Management After Ruptured Abdominal Aortic Aneurysms
Source: J Endovasc Ther. 2022 Mar 21;30(3):419–32. doi: 10.1177/15266028221083460 (PMC10209502; doi:10.1177/15266028221083460)
Supplement: sj-docx-4-jet-10.1177_15266028221083460 – Supplemental material for Nationwide Outcomes of Octogenarians Following Open or Endovascular Management After Ruptured Abdominal Aortic Aneurysms [file sj-docx-4-jet-10.1177_15266028221083460.docx]

**Supplementary Table 4**

**Overview of complications included in cardiac and abdominal complications**

Cardiac complications: myocardial infarction, heart failure, cardiac rhythm disturbances, other cardiac complications

Abdominal complications: abdominal abscess, abdominal sepsis, ileus, spleen injury, bowel ischemia, bowel injury, stoma placement, other abdominal complications.
