# Supplementary material for: Nationwide Outcomes of Octogenarians Following Open or Endovascular Management After Ruptured Abdominal Aortic Aneurysms
Source: J Endovasc Ther. 2022 Mar 21;30(3):419–32. doi: 10.1177/15266028221083460 (PMC10209502; doi:10.1177/15266028221083460)
Supplement: sj-docx-5-jet-10.1177_15266028221083460 – Supplemental material for Nationwide Outcomes of Octogenarians Following Open or Endovascular Management After Ruptured Abdominal Aortic Aneurysms [file sj-docx-5-jet-10.1177_15266028221083460.docx]

**Supplementary Table 5**

**Univariable and multivariable logistic regression analyses using original data completed by multiple imputation for EVAR and OSR to assess the association of patient-related risk factors with perioperative mortality in octogenarians**

| **EVAR** | **Univariable analysis** | | | **Multivariable analysis** | | |
| --- | --- | --- | --- | --- | --- | --- |
| **Factor** | **OR** | **95%-CI** | **P-value** | **aOR** | **95%-CI** | **P-value** |
| Age (per year) | 1.12 | 1.11 – 1.13 | <0.001 | 1.13 | 1.12 – 1.14 | <0.001 |
| Female sex | 0.75 | 0.69 – 0.81 | <0.001 | 0.69 | 0.63 – 0.75 | <0.001 |
| Cardiac comorbidity | 2.16 | 2.03 – 2.29 | <0.001 | 1.93 | 1.80 – 2.06 | <0.001 |
| Pulmonary comorbidity | 2.12 | 2.00 – 2.24 | <0.001 | 1.77 | 1.66 – 1.88 | <0.001 |
| Abnormalities on ECG | 2.22 | 2.09 – 2.35 | <0.001 | 1.54 | 1.44 – 1.65 | <0.001 |
| Creatinine ≥ 190 | 2.51 | 2.32 – 2.73 | <0.001 | 2.31 | 2.11 – 2.53 | <0.001 |
| Systolic blood pressure (per 10 mmHg) | 0.89 | 0.88 – 0.90 | <0.001 | 0.90 | 0.89 – 0.91 | <0.001 |
| GCS <12 | 3.89 | 3.53 – 4.29 | <0.001 | 3.30 | 2.97 – 3.66 | <0.001 |
| Hemoglobin <5.6 | 1.40 | 1.30 – 1.51 | <0.001 | 1.12 | 1.04 – 1.22 | 0.006 |
| Aortoiliac location | 0.69 | 0.62 – 0.77 | <0.001 | 0.62 | 0.55 – 0.69 | <0.001 |
| Diameter (per 10 mm) | 1.03 | 1.02 – 1.05 | <0.001 | 0.99 | 0.97 – 1.01 | 0.209 |
| **OSR** | **Univariable analysis** | | | **Multivariable analysis** | | |
| **Factor** | **OR** | **95%-CI** | **P-value** | **aOR** | **95%-CI** | **P-value** |
| Age (per year) | 1.00 | 0.99 – 1.01 | 0.672 | 1.00 | 0.99 – 1.01 | 0.451 |
| Female sex | 1.05 | 1.00 – 1.11 | 0.059 | 1.03 | 0.98 – 1.09 | 0.267 |
| Cardiac comorbidity | 1.76 | 1.68 – 1.85 | <0.001 | 1.85 | 1.75 – 1.95 | <0.001 |
| Pulmonary comorbidity | 1.42 | 1.34 – 1.49 | <0.001 | 1.36 | 1.29 – 1.44 | <0.001 |
| Abnormalities on ECG | 1.45 | 1.38 – 1.52 | <0.001 | 1.10 | 1.04 – 1.16 | <0.001 |
| Creatinine ≥ 190 | 0.64 | 0.58 – 0.71 | <0.001 | 0.64 | 0.58 – 0.71 | <0.001 |
| Systolic blood pressure (per 10 mmHg) | 0.90 | 0.89 – 0.90 | <0.001 | 0.89 | 0.88 – 0.90 | <0.001 |
| GCS <12 | 2.60 | 2.42 – 2.80 | <0.001 | 2.80 | 2.60 – 3.03 | <0.001 |
| Hemoglobin <5.6 | 0.88 | 0.82 – 0.94 | <0.001 | 0.63 | 0.58 – 0.67 | <0.001 |
| Aortoiliac location | 1.00 | 0.82 – 1.21 | 1.000 | - |  |  |
| Diameter (per 10 mm) | 0.99 | 0.98 – 1.00 | 0.065 | 1.00 | 0.99 – 1.01 | 0.841 |
