# Supplementary material for: Nationwide Outcomes of Octogenarians Following Open or Endovascular Management After Ruptured Abdominal Aortic Aneurysms
Source: J Endovasc Ther. 2022 Mar 21;30(3):419–32. doi: 10.1177/15266028221083460 (PMC10209502; doi:10.1177/15266028221083460)
Supplement: sj-docx-6-jet-10.1177_15266028221083460 – Supplemental material for Nationwide Outcomes of Octogenarians Following Open or Endovascular Management After Ruptured Abdominal Aortic Aneurysms [file sj-docx-6-jet-10.1177_15266028221083460.docx]

**Supplementary Table 6**

**Univariable and multivariable logistic regression analyses using original data completed by multiple imputation for EVAR and OSR to assess the association of patient-related risk factors with major complications in octogenarians**

| **EVAR** | **Univariable analysis** | | | **Multivariable analysis** | | |
| --- | --- | --- | --- | --- | --- | --- |
| **Factor** | **OR** | **95%-CI** | **P-value** | **aOR** | **95%-CI** | **P-value** |
| Age (per year) | 1.09 | 1.08 – 1.10 | <0.001 | 1.08 | 1.07 – 1.09 | <0.001 |
| Female sex | 0.66 | 0.62 – 0.71 | <0.001 | 0.68 | 0.63 – 0.73 | <0.001 |
| Cardiac comorbidity | 1.70 | 1.61 – 1.80 | <0.001 | 1.43 | 1.35 – 1.52 | <0.001 |
| Pulmonary comorbidity | 2.00 | 1.89 – 2.12 | <0.001 | 1.67 | 1.58 – 1.78 | <0.001 |
| Abnormalities on ECG | 2.10 | 1.98 – 2.21 | <0.001 | 1.60 | 1.51 – 1.70 | <0.001 |
| Creatinine ≥ 190 | 2.47 | 2.26 – 2.71 | <0.001 | 2.19 | 1.99 – 2.41 | <0.001 |
| Systolic blood pressure (per 10 mmHg) | 0.92 | 0.92 – 0.93 | <0.001 | 0.93 | 0.92 – 0.94 | <0.001 |
| GCS <12 | 4.34 | 3.86 – 4.90 | <0.001 | 3.70 | 3.28 – 4.19 | <0.001 |
| Hemoglobin <5.6 | 1.27 | 1.18 – 1.37 | <0.001 | 1.03 | 0.95 – 1.12 | 0.494 |
| Aortoiliac location | 1.01 | 0.92 – 1.12 | 0.788 | - |  |  |
| Diameter (per 10 mm) | 1.04 | 1.02 – 1.05 | <0.001 | 1.02 | 1.00 – 1.03 | 0.059 |
| **OSR** | **Univariable analysis** | | | **Multivariable analysis** | | |
| **Factor** | **OR** | **95%-CI** | **P-value** | **aOR** | **95%-CI** | **P-value** |
| Age (per year) | 0.97 | 0.96 – 0.98 | <0.001 | 0.96 | 0.96 – 0.97 | <0.001 |
| Female sex | 1.27 | 1.20 – 1.34 | <0.001 | 1.29 | 1.21 – 1.37 | <0.001 |
| Cardiac comorbidity | 1.43 | 1.36 – 1.51 | <0.001 | 1.40 | 1.32 – 1.48 | <0.001 |
| Pulmonary comorbidity | 1.27 | 1.20 – 1.34 | <0.001 | 1.23 | 1.16 – 1.30 | <0.001 |
| Abnormalities on ECG | 1.46 | 1.39 – 1.54 | <0.001 | 1.25 | 1.18 – 1.32 | <0.001 |
| Creatinine ≥ 190 | 0.69 | 0.63 – 0.76 | <0.001 | 0.66 | 0.59 – 0.73 | <0.001 |
| Systolic blood pressure (per 10 mmHg) | 0.92 | 0.91 – 0.92 | <0.001 | 0.92 | 0.92 – 0.93 | <0.001 |
| GCS <12 | 3.11 | 2.84 – 3.42 | <0.001 | 2.96 | 2.69 – 3.26 | <0.001 |
| Hemoglobin <5.6 | 1.44 | 1.34 – 1.55 | <0.001 | 1.18 | 1.09 – 1.27 | <0.001 |
| Aortoiliac location | 0.94 | 0.77 – 1.15 | 0.532 | - |  |  |
| Diameter (per 10 mm) | 1.02 | 1.01 – 1.03 | 0.007 | 1.05 | 1.03 – 1.06 | <0.001 |
